# The Role of Mitochondrial DNA Variation in Drug Response: A Systematic Review

**DOI:** 10.3389/fgene.2021.698825

**Published:** 2021-08-17

**Authors:** Samantha W. Jones, Amy L. Ball, Amy E. Chadwick, Ana Alfirevic

**Affiliations:** ^1^Department of Pharmacology and Therapeutics, MRC Centre for Drug Safety Science, University of Liverpool, Liverpool, United Kingdom; ^2^Department of Pharmacology and Therapeutics, Wolfson Centre for Personalised Medicine, University of Liverpool, Liverpool, United Kingdom

**Keywords:** mitochondrial DNA, haplogroup, drug, response, efficacy, toxicity, resistance

## Abstract

**Background:** The triad of drug efficacy, toxicity and resistance underpins the risk-benefit balance of all therapeutics. The application of pharmacogenomics has the potential to improve the risk-benefit balance of a given therapeutic via the stratification of patient populations based on DNA variants. A growth in the understanding of the particulars of the mitochondrial genome, alongside the availability of techniques for its interrogation has resulted in a growing body of literature examining the impact of mitochondrial DNA (mtDNA) variation upon drug response.

**Objective:** To critically evaluate and summarize the available literature, across a defined period, in a systematic fashion in order to map out the current landscape of the subject area and identify how the field may continue to advance.

**Methods:** A systematic review of the literature published between January 2009 and December 2020 was conducted using the PubMed database with the following key inclusion criteria: reference to specific mtDNA polymorphisms or haplogroups, a core objective to examine associations between mtDNA variants and drug response, and research performed using human subjects or human *in vitro* models.

**Results:** Review of the literature identified 24 articles reporting an investigation of the association between mtDNA variant(s) and drug efficacy, toxicity or resistance that met the key inclusion criteria. This included 10 articles examining mtDNA variations associated with antiretroviral therapy response, 4 articles examining mtDNA variants associated with anticancer agent response and 4 articles examining mtDNA variants associated with antimicrobial agent response. The remaining articles covered a wide breadth of medications and were therefore grouped together and referred to as “other.”

**Conclusions:** Investigation of the impact of mtDNA variation upon drug response has been sporadic to-date. Collective assessment of the associations identified in the articles was inconclusive due to heterogeneous methods and outcomes, limited racial/ethnic groups, lack of replication and inadequate statistical power. There remains a high degree of idiosyncrasy in drug response and this area has the potential to explain variation in drug response in a clinical setting, therefore further research is likely to be of clinical benefit.

## Introduction

Drug toxicity, in the form of adverse drug reactions (ADRs), represents a significant encumbrance to healthcare providers and the pharmaceutical industry alike. From a clinical standpoint, ADRs are associated with increased patient morbidity and mortality, non-adherence to medications and prolonged hospital stays (Sultana et al., 2013). In economic terms, it is estimated that ADRs are responsible for the attrition of ~30% of candidate pharmaceuticals, contributing to modern day research and development outlay estimates approaching $1.4 billion per new compound (Guengerich, 2011; DiMasi et al., 2016). The single most common cause of drug attrition, however, is a lack of efficacy, which alongside acquired resistance associated with antimicrobial agents, a number of chemotherapeutic, targeted oncogenic therapies, epilepsy and HIV drugs (Taylor et al., 2019; Vasan et al., 2019; Puertas et al., 2020), is a major healthcare issue. For example, it is estimated that antiepileptic medications fail to control seizures in 20–30% of patients (Sisodiya, 2005; Goldenberg, 2010).

The notion that genetic variation underpins variability in drug response was first proposed in the early twentieth century and forms the core principles of pharmacogenomics (Roden et al., 2011). Investigations into genetic predictors of drug efficacy have only identified a small number of clinically relevant associations to-date, for a comprehensive review [see Nelson et al. (2016)]. Genetic factors have also been closely associated with the onset of ADRs, with one of the best-known examples being the association between the human leukocyte antigen (HLA) allele B^*^57:01 and abacavir hypersensitivity (Hetherington et al., 2001; Mallal et al., 2008; Illing et al., 2017).

For the purpose of this review, the term “drug response” will refer to the efficacy, resistance or toxicity associated with a drug treatment. Drug efficacy is defined as the extent to which a drug has the ability to bring about its intended effect, drug toxicity is defined as the occurrence of adverse effects associated with drug treatment and drug resistance is defined as the reduction in the effectiveness of a drug. The authors acknowledge the interchangeability between a lack of efficacy and drug resistance in the literature, and note that drug resistance is the preferred term for a lack of response to antimicrobial, anticancer and antiepileptic treatments in particular.

To-date, investigations into the inter-individual variation underpinning drug response have largely focused upon the nuclear genome. However, eukaryotic cells have a second, often overlooked, source of genetic material; the mitochondrial genome. Indeed, our knowledge and understanding of the mitochondrial genome has rapidly expanded over the past two decades, providing the impetus to investigate its role in both human health and disease (Chinnery and Hudson, 2013).

Mitochondria are both structurally and functionally distinctive organelles; hosting unique features such as the electron transport chain (ETC), cardiolipin rich membranes and mitochondrial DNA (mtDNA), to name but a few. This makes mitochondria ideal candidates for off-target drug interactions, often leading to impaired function (Dykens and Will, 2007; Will and Dykens, 2014). Mitochondrial dysfunction has been increasingly implicated in a variety of drug-induced organ toxicities (Schirris et al., 2015; Varga et al., 2015; Ball et al., 2016; Hendriks et al., 2019), therefore it has been hypothesized that mtDNA variation, and by extension its phenotypic consequences, particularly from a bioenergetic standpoint, may underpin some of the idiosyncrasies associated with drug response (Boelsterli and Lim, 2007; Penman et al., 2020).

**Figure 1:**
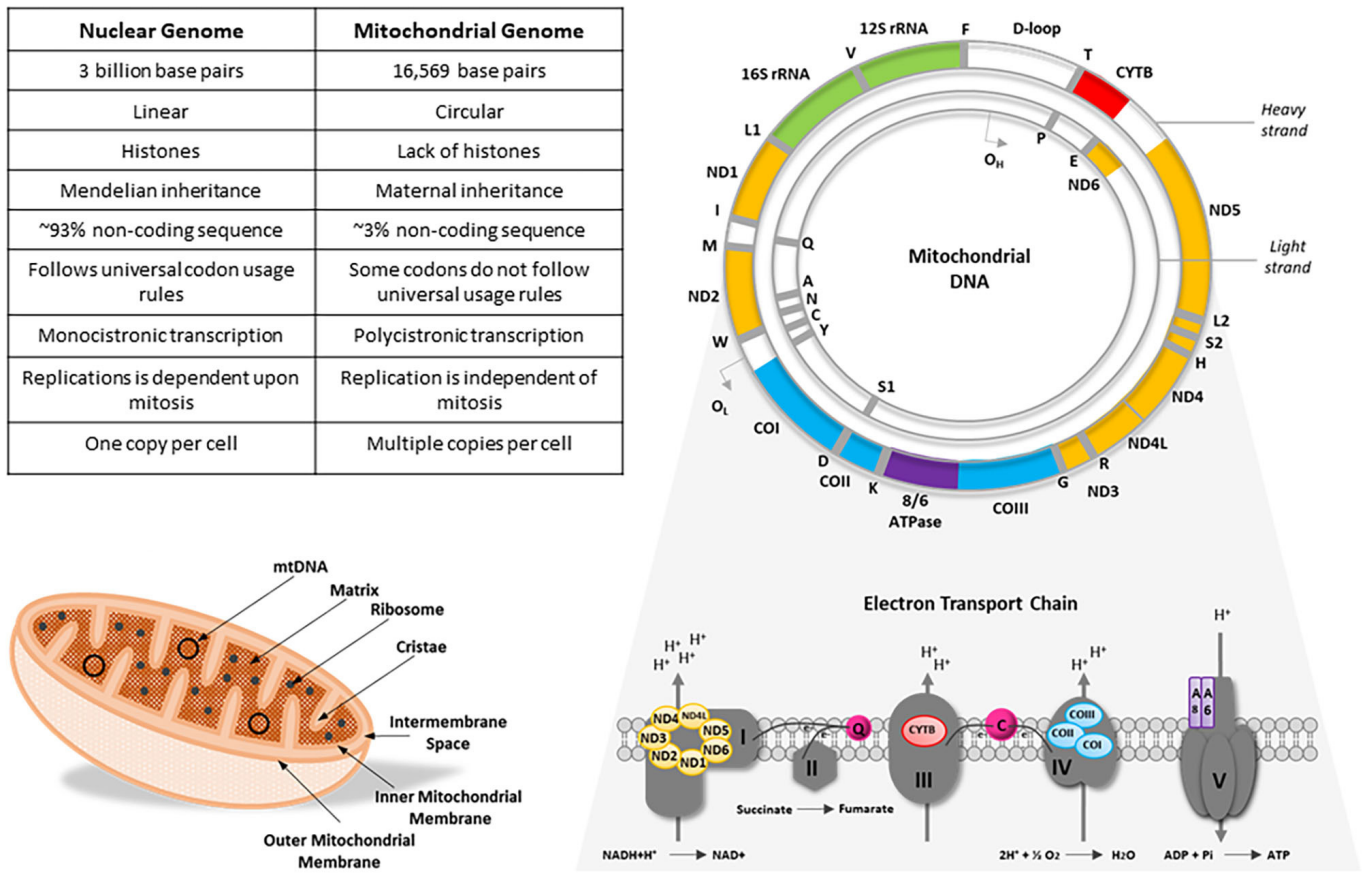
A comparison of the nuclear and mitochondrial genomes alongside an illustrative example of an mtDNA molecule and mitochondrion, with a schematic representation of the mitochondrial respiratory chain. Mitochondrial DNA is a circular, double-stranded molecule that encodes 13 integral subunits of the electron transport chain. Color-coded genes on the mtDNA molecule correspond to their cognate subunits on the electron transport chain. CO, cytochrome *c* oxidase; CYTB, cytochrome *b*; IMS, intermembrane space; ND, NADH dehydrogenase; O_H_, H-strand origin of replication; O_L_, L-strand origin of replication; Q, ubiquinone; rRNA, ribosomal RNA. Figure adapted from DiMauro and Schon (2003) and Penman et al. (2020).

Human mtDNA is ~16,569 base pairs in length and is arranged as a circular, double stranded DNA molecule. Mitochondrial DNA encodes a total of 37 genes; 13 genes code for integral structural subunits of the mitochondrial ETC, whilst the remainder encode 22 tRNA molecules, 16S ribosomal RNA and 12S ribosomal RNA (Chinnery and Hudson, 2013). Unlike its nuclear counterpart, mtDNA lacks intronic regions, possesses overlapping reading frames and only has one regulatory region, the displacement loop (D-loop), which provides a site of interaction for nuclear-encoded mtDNA replicative machinery (Chinnery and Hudson, 2013). It is widely accepted that in most multicellular organisms, mtDNA molecules are inherited exclusively from the maternal germline in a non-Mendelian fashion, however there are known exceptions to this dogma (DiMauro and Schon, 2003; Sato and Sato, 2013; Luo et al., 2018) (Figure 1).

Due to errors accrued during genome replication in tandem with a relatively low endogenous repair capacity, mtDNA molecules have an exceptionally high mutational rate, in the order of 10- to 17-fold higher than nuclear DNA genes of similar function (Wallace, 2000; Song et al., 2005; Larsson, 2010; Tuppen et al., 2010; Wallace and Chalkia, 2013). There are thought to be three clinically relevant types of mtDNA mutation: functional variants associated with ancient maternal lineages, recent deleterious, germ-line mutations that can result in maternally inheritable disease, or somatic mutations which accumulate in tissues over time (Wallace, 1994, 2013). Single nucleotide polymorphisms (SNPs) in mtDNA are often inherited together forming what are known as mitochondrial haplogroups. A haplogroup represents a group of similar and related descendant haplotypes that share a common ancestor but are distinguishable by the addition of functionally significant SNPs. Certain SNPs, and by extension haplogroups, have a high degree of association with certain disease states such as encephalomyopathy, deafness and myoclonic epilepsy (Goto et al., 1990; Shoffner et al., 1990; Nesbitt et al., 2013; Russell and Turnbull, 2014).

While specific SNPs may be correlated with mitochondrial dysfunction, there are several factors that affect the penetrance of a SNP. For example, there are multiple copies of mtDNA per cell, a state known as polyploidy. Although mtDNA copy number varies significantly from cell to cell (a factor which is largely dictated by energetic demand) the average copy number of mtDNA is estimated to be anywhere between 500–2000 in lung/liver cells and 4000–6000 in cardiac and skeletal muscle cells (D'Erchia et al., 2015; Reznik et al., 2016). However, the mere presence of a SNP does not guarantee the manifestation of phenotypic consequences, particularly where genome copy numbers are high.

While somatic tissue tends toward a state where polymorphisms present in mtDNA molecules are homoplasmic (i.e., present in all copies), mutations can affect a small subset of the mtDNA population, resulting in multiple distinct sequences which can be both inter- or intra-mitochondrial, a state known as heteroplasmy (Wallace and Chalkia, 2013). Therefore, propagation of bioenergetic defects requires a minimum critical ratio of mutant to wild-type mtDNA to be met, typically in the range of 60–90% (Tuppen et al., 2010).

An increased understanding of the unique vulnerabilities of the mitochondrial genome, coupled with progression in mtDNA sequencing capabilities, has paved the way for studies investigating mtDNA-dependent changes in drug response, yet to-date, there has not been a comprehensive review examining this role. Therefore, the objective of this article was to critically evaluate and summarize the available literature, across a defined period, in a systematic fashion in order to map out the current landscape of the subject area and identify how the field may continue to advance.

## Methods

### Search Strategy and Selection Criteria

All literature searches were performed using the MEDLINE (PubMed) database using the following standard filter settings: date range, January 2009-December 2020; text availability, full-text; article type, journal article. Non-English language publications were excluded from review. The selected search terms and search term combinations are outlined in Table 1.

**Table 1:** All searches were conducted using the MEDLINE (PubMed) database, with full-text papers published between 2009 and 2020 included.

**Search Terms**		
**1**	**2**	**3**
Mitochondrial haplogroup	Drug	Efficacy
MtDNA		Resistance
Mitochondrial genome		Response
		Toxicity

*Searches consisted of all combinations of search term categories 1, 2 and 3 e.g., “Mitochondrial haplogroup” AND “Drug” AND “Efficacy”*.

*mtDNA, mitochondrial DNA*.

**Figure 2:**
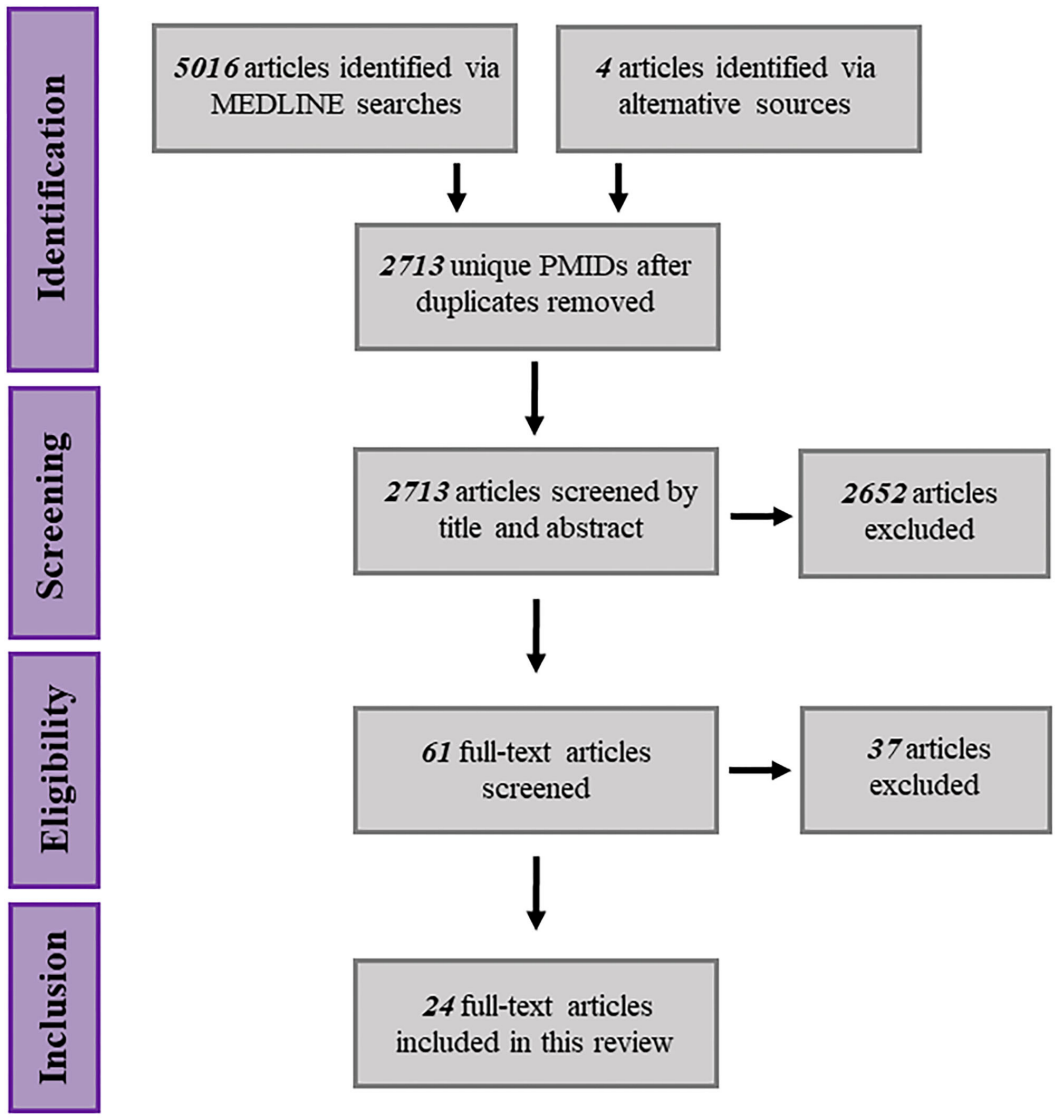
Schematic representation of the systematic search procedure, spanning article identification, eligibility screening and inclusion.

The Preferred Reported Items of Systematic Reviews and Meta-Analyses (PRISMA) checklist and flow diagram were used to guide this review (Figure 2) (Moher et al., 2009). Results gathered from all search combinations were reviewed manually by screening for relevant titles and abstracts. Four articles were identified through the “similar articles” feature on the PUBMED database and labeled as “articles identified through alternative sources.” Titles and abstracts that described mtDNA variation within the context of drug response were carried forward for full-text screening. Full-text screening was performed by using the following inclusion criteria: articles must refer to specific mtDNA polymorphisms and/or haplogroups, the core objective of the article must be to examine associations between mtDNA variants and drug response, the research must be performed using human subjects or human *in vitro* models. Possible reasons for subsequent article exclusion are summarized in Table 2.

**Table 2:** Summary of full-text article exclusion criteria with assigned numerical coding.

**Numerical Code**	**Reason for Exclusion**
1	Publication is not an original research article
2	Publication assesses the effect of mtDNA depletion rather than mtDNA variation
3	Publication uses non-human models
4	Publication assesses the effect of mtDNA variation on disease/health rather than drug response
5	Publication assesses the effect of mtDNA variation on chemical-induced rather than drug-induced toxicity
6	Other

*mtDNA, mitochondrial DNA*.

Finally, all authors were required to agree that articles carried forward for review met the inclusion criteria as stated above. Two authors (SJ and AB) reviewed included articles and collated study information into data abstraction tables. Uncertainties arising from compiled study data were resolved by author consensus decision.

## Results

### Article Inclusion Summary and Analysis of Baseline Demographics

After conducting initial screening and eligibility assessments, 24 full-text articles fulfilled the inclusion criteria for review. An abstraction table was generated in order to collate key demographic information from each publication including study size, disease indication, gender distribution, age distribution and ethnicity data (Table 3). In reference to the specific disease indications stated for each study, of the 24 articles included in the review; 10 (41.7%) were focussed upon human immunodeficiency virus (HIV) infection/acquired immunodeficiency syndrome (AIDs). Four articles were concerned with *in vitro* cancer research, specifically prostate, lung (small cell) and cisplatin-resistant cancers (various) (16.7%). Four articles were ascribed to bacterial infections research (16.7%), ranging from gram-positive infections to tuberculosis. The remaining six articles spanned the areas of age-related macular degeneration, migraines, schizophrenia, thromboembolic disease, hepatitis C infection and menopausal symptoms (4.2% each). Gender distribution across studies tended to skew toward a male majority with the exception of seven studies (Di Lorenzo et al., 2009; Kampira et al., 2013a,b; Subiabre et al., 2015; Esteban et al., 2019; Wang et al., 2019; Abedi et al., 2020), one of which was concerned with the management of menopausal symptoms and thus enrolled female participants exclusively (Wang et al., 2019). In terms of study design, with the exception of six studies that used in cytoplasmic hybrid (cybrid) *in vitro* models, the remaining 18 articles examined drug responses across patient cohorts. For a comprehensive review of cybrid models (see Wilkins et al., 2014).

**Table 3:** Summary of publications included in this review, including key demographic information extracted from each article: study size, disease indication, age distribution and ethnicity data.

**Author(s)**	**Study Size**	**Disease Indication, Gender**	**Gender (Male/Female)**	**Age (Years)**	**Ethnicity**
Abedi et al. (2020)	14 cybrid cell lines	Cancer	4/10	Mean: 42.4 ± 8.90	African, Hispanic, Asian
Canter et al. (2010)	156	HIV/AIDS	110/46	Median (range): Controls: 34 (17–66), Cases: 42 (19–64)	Black, Non-Hispanic
Di Lorenzo et al. (2009)	64	Migraines	15/49	Range: 18–65	European
Esteban et al. (2019)	91	Neovascular age-related macular degeneration	34/57	Mean: 76.9 ± 7.5	Unknown
Garrabou et al. (2017)	19	Gram-positive infection	11/8	Mean: 65.8 ± 15.6	European
Grady et al. (2011)	423	HIV/AIDS	349/74	Median (Range): 36 (17–72)	Non-Hispanic white, Non-Hispanic Black and Hispanic
Guzmán-Fulgencio et al. (2013)	275 cases (162 controls)	HIV/AIDS	208/67^*^	Median (25–75 percentile): 37.8 (32–45.1)	European/Eurasian
Guzmán-Fulgencio et al. (2014)	304	HIV/HCV co-infection	231/73	Median (25–75 percentile): 42 (25–60)	White European
Hendrickson et al. (2009)	410	HIV/AIDS	410/0	Mean: 43.8 ± 7.0	White European-American
Hulgan et al. (2011)	231	HIV/AIDS	211/20	Median (IQR): 39 (17–73)	White, Non-Hispanic
Hulgan et al. (2012)	104	HIV/AIDS	75/29	Median (Range): 37 (17–72)	Non-Hispanic Black
Kampira et al. (2013a)	215	HIV/AIDS	83/132	Median (IQR): 38 (32–46)	Malawian
Kampira et al. (2013b)	117	HIV/AIDS	44/73	Median (IQR): 37 (31–46)	Malawian
Lee et al. (2019)	76	Tuberculosis	40/36	Mean: DILI (58.7 ± 18.9), Non-DILI (55.5 ± 20.0)	Taiwanese
Ma et al. (2015)	2 SCLC cell lines	Small cell lung cancer	1/0	61	Caucasian
Medrano et al. (2018)	324 cases (141 controls)	HIV/AIDS	264/60^*^	Median (25–75 percentile): 41(34.7–49.2)	European
Micheloud et al. (2011)	248	HIV/HCV co-infection	189/59	Median (25–75 percentile): 41 (37.8–44.7)	European
Mittal et al. (2017)	74	Schizophrenia	61/13	Mean: 40 ± 11.98	European
Muyderman et al. (2012)	3 cybrid cell lines	Bacterial Infection	1 (female)/2 unknown	Unknown	European
Pacheu-Grau et al. (2013)	25 osteosarcoma 143B cybrid cell lines	Gram-Positive Infection	Unknown	Unknown	European
Patel et al. (2019)	14 cybrid cell lines	Cancer	9/5	Mean: Haplogroup H (30.57 ± 3.39), Haplogroup J (36.14 ± 5.47 years)	European
Sun et al. (2015)	1 patient (*n* = 3 wild type/mutant cybrid cell lines)	Prostate Cancer	1 (male)	Unknown	Unknown
Subiabre et al. (2015)	191	Thromboembolic Disease	77/114	Mean: Male: (65 ± 13), Female: (66 ± 13)	Amerindian Ancestry
Wang et al. (2019)	46	Menopausal Symptoms	0/46	Mean: Placebo 53.9 ± 3.14, PS 50 mg/day 53.8 ± 3.78, PS 100 mg/day 55.3 ± 2.83	Hispanic or Latino, Asian, African-American, White or 'Unknown'

* *Age demographics stated for cases only. AIDS, acquired immunodeficiency syndrome; DILI, drug-induced liver injury; HCV, hepatitis C virus; HIV, human immunodeficiency virus; IQR, interquartile range; PS, phytoSERM*.

When further examining the distribution of ethnic groups across studies, it was evident that the majority of articles examined either European or Caucasian populations exclusively (54.2%), or in cases where investigations spanned multiple ethnic groups, White “Non-Hispanic” subjects were included in the study (8.3%). In comparison, seven articles included black or African-American subjects (29.2%), three articles included Latino or Hispanic subjects (12.5%), three articles included Asian subjects (12.5%) and one article included those of Amerindian descent (4.2%). In the case of two publications, the ethnic origins of the study participants were not clearly stated (Sun et al., 2015; Esteban et al., 2019).

### Mitochondrial DNA Variants Associated With Altered Responses to Antiretroviral Therapy

Modern antiretroviral therapies (ARTs) have revolutionized the management of HIV infection across many settings. Indeed, early administration of ART has long been deemed essential for the optimisation of health outcomes at both the individual and population level (Hulgan and Gerschenson, 2012). However, ART regimens, particularly those incorporating older nucleoside reverse-transcriptase inhibitors (NRTIs) and more recently protease inhibitors (PIs), have been associated with both generalized and tissue-specific mitochondrial toxicities such as myopathy, lactic acidosis, lipoatrophy, metabolic syndrome and cardiovascular complications (Feeney and Mallon, 2010; Hulgan and Gerschenson, 2012).

Given the widely established link between ART regimens and drug-induced mitochondrial dysfunction, it was unsurprising that the majority of articles identified though systematic literature screening pertained to the effects of mtDNA variation upon response to antiretroviral medications (Table 4). Nonetheless, this link is complicated by the discovery that the mere presence of HIV polypeptides, in the absence of ART, results in the manifestation of mitochondrial perturbations and apoptosis (Muthumani et al., 2003; Roumier et al., 2003).

**Table 4:** Summary of the publications included in this review that report differential responses to antiretroviral medications.

**Author(s)**	**Study Description**	**Drug(s)**	**Sequencing Method**	**Outcome**	**Association**	**Odds Ratio/95% Confidence Interval/*p*-value**
Canter et al. (2010)	156 Non-Hispanic, Black participants	Didanosine and stavudine or zidovudine and lamivudine, with nelfinavir	Samples were sequenced using GeneChip® Human Mitochondrial Resequencing Array v2.0 Affymetrix, Inc., Santa Clara, CA, USA	Peripheral neuropathy-associated with NRTI treatment regimens	African mtDNA subhaplogroup L1c was an independent predictor of peripheral neuropathy	3.7/1.1–12.0/0.03
Grady et al. (2011)	423 HIV-1 infected, ART naïve patients	ART (didanosine and stavudine or zidovudine and lamivudine combined with either efavirenz, nelfinavir or both)	Full mtDNA sequencing was performed using the GeneChipR Human Mitochondrial Resequencing Array v2.0 (Affymetrix, Inc., Santa Clara, CA, USA)	ART efficacy in the recovery of CD4+ T-cells following HIV-infection	The most significant SNP associations were those tagging the African L2 haplogroup, which was associated with a decreased likelihood of ≥100 cells/mm3 CD4 count increase at week 48 in non-Hispanic blacks	0.17/0.06–0.53/0.002
Guzmán-Fulgencio et al. (2013)	275 cART-naïve patients with CD4^+^ counts <350 cells/mm^3^, who were followed-up at least 24 months after initiating cART	cART (focus on NRTIs)	Genotyping was performed using the Sequenom MassARRAY platform iPLEX® Gold assay design system	cART efficacy in the recovery of CD4^+^ T-cells following HIV-infection	Haplogroup H had a greater chance than haplogroups J and T of achieving CD4^+^ count ≥500 cells/mm^3^ during at least 48 and 60 months	48 months:J: 3.56/1.05–12.10/0.042T: 6.31/1.32–30.11/0.04460 months:J: 4.34/1.04–18.03/0.021T: 6.24/1.09–37.70/0.040
Hendrickson et al. (2009)	HIV-infected European American patients on HAART	HAART	Six haplotype-tagging SNPs were initially used to classify individuals as mitochondrial macro-haplogroups N, M and L. Haplogroups within the Western European (N) subset were further divided into haplogroups using SNPs in the MHCFV approach	HAART-induced lipoatrophy	Haplogroup H was associated with increased lipoatrophy on the arms, legs and buttocks, whilst haplogroup T was associated with marginal protection against lipoatrophy	H:Arms: 1.77/1.17–2.69/0.007Legs: 1.54/1.03–2.31/0.037Buttocks: 1.41/0.94–2.12/0.10T: 0.52/0.20–1.00/0.05
Hulgan et al. (2011)	231 White, Non-Hispanic participants	ART regimens (lopinavir, ritonavir, efavirenz, zidovudine, stavudine [XR formulation] or tenofovir)	Genotyping was performed with the ABI PRISM® 7900HT Sequence Detection System using the 5' nuclease allelic discrimination Taqman™ assay	mtDNA influences ART complications	Compared with other haplogroups, haplogroup I had more baseline extremity fat and more extremity fat loss by DEXA (−13% [−13 to 12] vs. +9% [−13 to 26]) and lipoatrophy (50 vs. 20%)	3.9/1.1–14.4/0.04
Hulgan et al. (2012)	104 Non-Hispanic, Black participants	ART (didanosine and stavudine or zidovudine and lamivudine combined with either efavirenz, nelfinavir or both)	MtDNA sequencing was performed using the GeneChip® Human Mitochondrial Resequencing Array v2.0 (Affymetrix, Inc., Santa Clara, CA, USA). Haplogroups were assigned using Hernstadt classification	ART efficacy in the recovery of CD4^+^ T-cells following HIV infection	Compared with non-L2 haplogroups, subjects in haplogroup L2 had lower baseline activated CD4^+^ cells (median: 12% vs. 17%). At 48 weeks of ART, median change from baseline was significantly less among L2 vs. non-L2 haplogroups (+95 cells vs. +178)	NR/NR/0.03NR/NR/0.002
Kampira et al. (2013a)	215 patients on stavudine-containing ART	Stavudine	Samples were sequenced from nucleotide position 577–15,953 according to the rCRS. Capillary electrophoresis for sequencing reactions was run on an ABI PRISM, × l Genetic Analyzer	Stavudine-induced peripheral neuropathy	mtDNA subhaplogroup L0a2 was independently associated with an increased risk of peripheral neuropathy	2.23/1.14–4.39/0.019
Kampira et al. (2013b)	117 adult HIV/AIDS patients on stavudine-containing ART	ART (stavudine, lamivudine and nevirapine)	Samples were sequenced from nucleotide position 577–15,953 according to the rCRS. Capillary electrophoresis for sequencing reactions was run on an ABI PRISM 3,130 × l Genetic Analyzer	ART-induced lipodystrophy	Subhaplogroup L3e appeared to protect patients against lipodystrophy	NR/NR/NR
Medrano et al. (2018)	324 naïve cART patients with CD4^+^ <200 cells/mm^3^, who were followed up during the 24 months after initiating cART	Cart (PIs, NNRTIs, PIs and NNRTIs or “other”)	Samples were genotyped by using iPLEX® Gold technology and the Agena Bioscience MassARRAY platform (San Diego, CA, USA)	cART efficacy in the recovery of CD4^+^ T-cells following HIV infection	Higher frequency of H haplogroups in patients with high CD4^+^ recovery. Patients of haplogroup H had greater odds of having a better CD4^+^ T-cell recover than non-H haplogroups	1.75 (adjusted)/1.04–2.95/0.035NR/NR/0.032
Micheloud et al. (2011)	248 HIV/HCV–coinfected patients on HAART	HAART	MtDNA genotyping was performed using the Sequenom MassARRAY platform	Metabolic disorders and cardiovascular diseases in HIV/HCV–coinfected patients on HAART	Haplogroups HV and H had reduced odds of insulin resistance and high atherogenic risk. Haplogroups U had increased odds of insulin resistance. Haplogroups JT and T had increased risk of high atherogenic index	Insulin resistance:HV: 0.45/0.24–0.85/0.013H: 0.36/0.18–0.69/0.002U: 2.66/1.39–5.08/0.003Atherogenic index:HV: 0.44/0.22–0.87/0.019H: 0.40/0.19–0.80/0.011JT: 2.86/1.29–6.33/0.009T: 4.01/1.59–10.03/0.003

*Abstraction table details the author(s), study design, drug(s) of interest, sequencing methods, outcomes, associations and statistics. ACTG, AIDS Clinical Trials Group; (c)ART, (combined) antiretroviral therapy; DEXA, dual-energy X-ray absorptiometry; HAART, highly active antiretroviral therapy; HCV, hepatitis C virus; HIV, human immunodeficiency virus; MHCFV, mitochondrial haplogrouping using candidate functional variants approach; mtDNA, mitochondrial DNA; NR, not reported; (N)NRTIs, (non)-nucleotide/nucleoside reverse transcription inhibitors; PI, protease inhibitors; rCRS, revised Cambridge reference sequence; SNP, single nucleotide polymorphism*.

### Mitochondrial DNA Variants and Antiretroviral Therapy Efficacy

As outlined in Table 4, four studies examined the effects of ART efficacy in relation to the recovery of CD4^+^ T-lymphocyte count following HIV-infection. CD4^+^ lymphocyte count is a key determinant of disease progression amongst HIV-infected individuals, however, there is substantial inter-individual variation in both the rate and magnitude of CD4^+^ recovery after the initiation of ART (Grady et al., 2011). This may be partially underpinned by genetic variation, particularly functional variation in mtDNA, modulating the T-cell response to exogenous stressors (ART and/or HIV) (Grady et al., 2011).

In a study conducted by Grady et al. (2011), full mitochondrial genome sequencing data from the U.S.-based AIDS Clinical Trial Group (ACTG) was used to determine if there were associations between functional mtDNA variants and CD4^+^ lymphocyte count increase ≥100 cells/mm^3^ at 48 weeks of follow-up. The most robust association with CD4^+^ count change at 48 weeks was amongst the Non-Hispanic Black participants. Specifically, polymorphisms that defined mitochondrial haplogroup L2 (base positions: 2789, 7175, 7274, 7771, 9221, 10115, 11914, 13590, 13803, 14566 and 16390 (*p*-value range = 0.002–0.027) were associated with decreased odds of CD4^+^ count change of ≥100 cells/mm^3^.

In a follow-up study using the ACTG patient cohort, investigators examined whether additional T-lymphocyte parameters differed by mtDNA haplogroup in Non-Hispanic Black participants. When comparing mtDNA haplogroup L2 with non-L2 haplogroups, the baseline median percentage of activated (CD38^+^/HLA-DR^+^) CD4^+^ lymphocytes was significantly lower (12% [IQR 6, 22] vs. 17%; *p* = 0.03) in patients belonging to the L2 haplogroup. After 48 weeks ART, the median change from baseline was significantly less amongst the L2 haplogroup patients (+95 [−3, +182] cells) compared with patients in non-L2 haplogroups (+178 [+105, +312] cells; *p* = 0.002) (Hulgan et al., 2012).

The remaining two articles examined whether European haplogroups or major haplogroup clusters may influence CD4^+^ T-lymphocyte recovery in HIV-infected patients following combination ART (cART) (Guzmán-Fulgencio et al., 2013; Medrano et al., 2018). Guzmán-Fulgencio et al. (2013) showed that patients belonging to haplogroup cluster JT and haplogroup J had a lower chance of achieving a CD4^+^ count ≥ 500 cells/mm^3^ than those belonging to cluster HV (hazard ratio [HR] = 0.68; *p* = 0.058) or haplogroup H (HR = 0.48; *p* = 0.010). Furthermore, cluster HV and haplogroup H had a greater chance of achieving CD4^+^ count ≥500 cells/mm^3^ at 12, 36, 48 and 60 months post-cART initiation. In-line with this, Medrano et al. (2018) showed that there was a higher frequency of patients belonging to haplogroup H with high CD4^+^ recovery (≥ 9.65 CD4^+^ cells/mm^3^/month; *p* = 0.032). Additionally, patients belonging to haplogroup H had higher odds of having a better CD4^+^ recovery than patients with non-H haplogroups (*p* = 0.035).

### Mitochondrial DNA Variants and Antiretroviral Therapy-Associated Toxicity

Antiretroviral therapies or more specifically NRTIs, have been associated with complications related to mitochondrial dysfunction in their recipients. Cases of distal sensory peripheral neuropathy have been known to develop in ART-treated persons exposed to NRTIs, particularly didanosine, stavudine and dideoxycytidine (Dalakas, 2001; Dalakas et al., 2001). In a study conducted by Canter et al. (2010) using the ACTG cohort, it was found that African subhaplogroup L1c was independently associated with the development of peripheral neuropathy in Non-Hispanic Black participants treated with NRTIs (*p* = 0.03) (Table 4). A subsequent study carried out by Kampira et al. (2013a) investigated mtDNA haplogroups and their role in susceptibility to stavudine-associated peripheral neuropathy in a Malawian patient cohort. The results showed that the African L0a2 subhaplogroup was an independent risk factor for the development of peripheral neuropathy (*p* = 0.019), whilst subhaplogroup L2a was associated with reduced risk (*p* = 0.036).

Development of metabolic complications such as dyslipidemia, abnormal lipid accumulation and peripheral lipoatrophy are another prominent risk associated with ART regimens. Links between European mtDNA haplogroup I and metabolic changes have been identified in White, Non-Hispanic participants within in the ACTG patient cohort. Compared with other haplogroups, haplogroup I participants tended to have higher extremity fat at baseline and greater extremity fat loss by whole-body dual-energy X-ray absorptiometry (DEXA; −13% [−31 to +12] vs. +9% [−13 to +26]; *p* = 0.08) and lipoatrophy (50% vs. 20%; *p* = 0.04) (Hulgan et al., 2011) (Table 4). Conversely, in a Multicentre AIDS (MACS) cohort study consisting of HIV-1 infected Caucasian men, haplogroup H was strongly associated with significant extremity fat loss in the arms (*p* = 0.007) and legs (*p* = 0.03), whilst there was evidence to suggest that haplogroup T may be protective against lipoatrophy (*p* = 0.05) (Hendrickson et al., 2009).

When examining stavudine-associated lipodystrophy in a Malawian HIV/AIDS cohort, it was noted that none of the individuals belonging to mtDNA subhaplogroup L3e had lipodystrophy, thus the authors suggested that subhaplogroup L3e might be protective. During univariate analysis, subhaplogroup L2a had also shown a degree of association with lipodystrophy (*p* = 0.082) (Kampira et al., 2013b). However, the study was limited in regards to its statistical power and the definition of lipodystrophy without the use of DEXA to discriminate between lipoatrophy and lipid accumulation.

The final study conducted by Micheloud et al. (2011) was concerned with identifying associations between European mtDNA haplogroups and metabolic disorders in HIV/HCV co-infected patients on highly active antiretroviral therapy (HAART). It was shown that haplogroup cluster HV and haplogroup H had reduced odds of insulin resistance and atherogenic risk. Conversely, haplogroup U had increased odds of insulin resistance, whilst haplogroup cluster JT and haplogroup T had increased odds of atherogenic risk.

### Mitochondrial DNA Variants Associated With Altered Responses to Anticancer Agents

Chemotherapeutic agents, particularly when combined with other combative techniques such as radiotherapy and surgery, have dramatically improved survival rates in cancer patients. However, multi-drug resistance (MDR) presents a major clinical hurdle in regards to improving long-term patient survival outcomes (Ma et al., 2015; Mansoori et al., 2017).

A study conducted by Ma et al. (2015) investigated mtDNA sequences from the small cell lung cancer (SCLC) cell line, H446, and its drug-resistant counterpart H446/CDDP, comparing them to the revised Cambridge reference sequence (rCRS) in order to ascertain whether mtDNA mutations play a role in acquired drug resistance to cisplatin (Table 5). Both the H446 and H446/CDDP cell lines belonged to haplogroup G and harbored several point mutations that led to amino acid changes in mtDNA-encoded proteins (COI, ND3, ND5, cytb) and base shifts in rRNA. However, the sequences in the two cell lines were identical, therefore, mtDNA variation was not related to drug-resistance but more likely tumorigenesis.

**Table 5:** Summary of the publications included in this review that report differential responses to anticancer medications.

**Author(s)**	**Study Description**	**Drug(s)**	**Sequencing Method**	**Outcome**	**Association**	***p*-value(s)^a^**
Abedi et al. (2020)	APRE-19 cybrids belonging to haplogroups L, D and [A+B]	Cisplatin	NGS sequencing was performed as described by Patel et al. (2019)	Differential response to cisplatin treatment	Compared to their untreated controls: haplogroup L cybrids saw a greater decline in metabolic activity following cisplatin treatment (27.4%) than haplogroups D (24.86%) and [A+B] (24.67%), ALK expression decreased in haplogroups L and [A+B] but not haplogroup D, EGFR expression decreased in haplogroup [A+B] cybrids but not haplogroups L or D	NR
Ma et al. (2015)	SCLC cell line H446 and the multi-drug-resistant cell line H446/CDDP	Cisplatin	MtDNA was amplified using 26 primers, the PCR products were purified and sequenced by ABI Prism 3,700 instrument	SCLC cell line resistance to cisplatin treatment	mtDNA mutations were responsible for the tumorigenesis of SCLC but were not associated with the drug sensitivity of SCLC cell lines. Drug sensitive and drug resistant cell lines both belonged to haplogroup G	NR
Patel et al. (2019)	APRE-19 cybrids belonging to haplogroups H and J	Cisplatin	NGS sequencing was performed using 96 libraries and run on an Illumina HiSeq instrument	Differential response to cisplatin treatment	1) Haplogroup J cybrids were more sensitive to cisplatin treatment than haplogroup H cybrids in terms of cell viability 2) Cisplatin-treated haplogroup J cybrids showed significant reduction in ΔΨ_m_ compared with haplogroup H 3) Treated haplogroup J cybrids showed lower ROS than treated haplogroup H cybrids	1) 0.001 2) 0.0005 3) 0.006
Sun et al. (2015)	PC3 cybrid cell lines containing either wild type or an mtDNA COI gene mutation m.6124CT>C (Met74Thr)	Simvastatin	A mutation in the COI gene was identified as described in Arnold et al. (2013)	PC3 cybrid resistance to statin-induced cell death	PC3 cybrids with the m.6124CT > C mtDNA mutation were more resistant to simvastatin-induced apoptosis treatment than wild-type PC3 cybrids	0.024

*Abstraction table details the author(s), study design, drug(s) of interest, sequencing methods, outcomes, associations and statistics*.

a *Odds ratio and 95% confidence intervals were unavailable for these articles*.

*ALK, anaplastic lymphoma receptor tyrosine kinase; COI, cytochrome c; cybrid, cytoplasmic hybrid; ΔΨm, mitochondrial membrane potential, D-loop, displacement loop; EGFR, epidermal growth factor receptor; mtDNA, mitochondrial DNA; NGS, next-generation sequencing; NR, not reported; PCR, polymerase chain reaction; ROS, reactive oxygen species; SCLC, small cell lung cancer*.

In contrast, data gathered from Sun et al. (2015) indicated that the m.6124CT>C mutation (Met74Thr) in the mtDNA COI gene was associated with resistance to statin-induced apoptosis in the PC3 mutant cybrid line (wild type PC3 cybrids IC_50_ 0.28 ± 0.04 μM vs. mutant PC3 cybrids IC_50_ 2.12 ± 0.52 μM; *p* ≤ 0.024), primarily via the evasion of extrinsic apoptosis. The authors suggested that inherited mtDNA mutations may limit the beneficial effects of statin therapy in some prostate cancer patients due to drug resistance.

Aside from drug resistance, chemotherapeutic agents are often associated with the manifestation of ADRs. One such reaction affiliated with cisplatin treatment regimens is mild to moderate pigmentary retinopathy. In a study conducted by Patel et al. (2019), the authors demonstrated that *in vitro*, human retinal pigment epithelial (ARPE-19) cybrids respond differentially to cisplatin treatment depending upon their mitochondrial haplogroup (Table 5). Haplogroup J cybrids exposed to 25 and 50 μM cisplatin showed greater loss of cell viability (35%; *p* = 0.04 and 65%; *p* = 0.002, respectively), loss of mitochondrial membrane potential (75.9 ± 3.6%; *p* = 0.0001) and lower levels of reactive oxygen species (ROS; 56.79 ± 7.731%, *p* = 0.03) than both their untreated counterparts and haplogroup H cybrids. It was proposed that differences between H vs. J GG stretch patterns in the mtDNA D-loop may lead to differential formation of cisplatin-mtDNA adducts. Therefore, differential responses to cisplatin, mediated by mitochondrial genotype, may partially contribute to the development of retinopathies in susceptible patients.

A later study performed by Abedi et al. (2020) examined the effects of cisplatin exposure upon APRE-19 cybrids from non-European maternal lineages, specifically African haplogroup L, Hispanic macro-haplogroup cluster A+B and Asian haplogroup D. Haplogroup L cybrids exposed to 40 μM cisplatin showed the greatest reduction in metabolic activity (measured using tetrazolium dye [MTT] assay) compared with their untreated controls (75.0 ± 6.7% vs. 102.4 ± 7.0%; *p* = 0.0117), although cisplatin-treated haplogroup D and A+B cybrids also showed significant reductions in metabolic activity when compared against their respective controls (86.9 ± 1.6% vs. 111.7 ± 4.3%; *p* = 0.0001 and 70.3 ± 4.5% vs. 95.0 ± 8.5%; *p* = 0.0285). Post-cisplatin exposure, transcription levels of *ALK* in haplogroup L and A+B cybrids reduced significantly (fold change 0.18 ± 0.097; *p* <0.0001 and 0.17 ± 0.095; *p* = 0.0001, respectively) as did *EGFR* expression levels in haplogroup A+B cybrids (fold change 0.50 ± 0.1; *p* = 0.0246). The authors concluded that mtDNA haplogroups representing different racial/ethnic groups, may play a role in both cisplatin resistance and the manifestation of adverse events in patients.

### Mitochondrial DNA Variants Associated With Altered Responses to Antimicrobial Agents

Antibiotics that target ribosomes, such as linezolid, are used to combat pathogenic gram-positive bacterial infections and are known to elicit severe ADRs in their recipients, ranging from lactic and metabolic acidosis to myelosuppression leading to hematological disturbances (Garrabou et al., 2017). Whilst the mechanisms underpinning these adverse reactions are likely multifactorial, genetic variation may be a contributing factor (Pacheu-Grau et al., 2013). A study conducted by Garrabou et al. (2017) aimed to gain an understanding of clinical heterogeneity in response to identical linezolid exposures in a European patient cohort (Table 6). The data indicated that mitochondrial haplogroup H was associated with reduced linezolid toxicity (26.11% enhanced mitochondrial protein synthesis and 40.67% fewer clinical events) whilst haplogroup U trended toward increased toxicity (30.49% decreased mitochondrial protein synthesis and 67% more clinical events). However, these associations were not statistically significant. Polymorphisms in 12S rRNA and the m.2706A>G, m.3197T>C, and m.3010G>A polymorphisms in 16S rRNA trended toward an association with mitochondrial dysfunction and adverse clinical outcomes (0.05 < *p* < 0.12).

**Table 6:** Summary of the publications included in this review that report differential responses to antimicrobial medications.

**Author(s)**	**Study Description**	**Drug(s)**	**Sequencing Method**	**Outcome**	**Association**	**Odds Ratio/95% Confidence Interval/*p*-value**
Garrabou et al. (2017)	19 patients receiving linezolid for 1 month (600 mg/12 h) prospectively included. Patients received concomitant rifampin treatment (600 mg/day)	Linezolid	Mitochondrial Haplogroups determined by qPCR. Three SNPs defining haplogroups JT, H and U were genotyped. Fourteen other mtDNA SNPs were characterized using a phylogenetic approach. The Sanger methodology was used to sequence the MT-RNR1 and MT-RNR2 genes	Adverse clinical outcomes from linezolid treatment	Trend toward reduced linezolid toxicity in patients with Haplogroup H. Haplogroup U patients had increased mitotoxicity and more clinical symptoms. 16S rRNA m.3010G>A polymorphism enhanced trend toward toxicity	NR/NR/0.05 < *p* <0.12
Lee et al. (2019)	38 patients with TB who had experienced DILI (16, pyrazinamide; 14 rifampin; 8 isoniazid). 38 sex- and age-matched, non-DILI TB positive controls	Pyrazinamide, rifampin, isoniazid	Next generation sequencing using PacBio SMRT sequencing to detect point mutations across the entire mitochondrial genome	Anti-TB drug associated DILI	1) More mtDNA variants in patients with isoniazid-induced DILI vs. non-DILI 2) More mtDNA variants in gene *ND5* in patients with isoniazid-induced DILI vs. non-DILI 3) In gene *ND1*, the average number of mtDNA variants in patients with isoniazid-induced DILI was higher than that of the non-DILI group. 4) Greater number of non-synonymous substitutions in *ND5* were found in isoniazid-induced DILI cases 5) There were more private *ND5* polymorphisms in DILI vs. non-DILI groups	1) NR/NR/0.005 2) NR/NR/0.028 3) NR/NR/0.025 4) NR/NR/0.033 5) NR/NR/0.024
Muyderman et al. (2012)	Lymphoblastoid cybrids generated from a South Italian individual harboring the m.1095T>C mutation in the 12S rRNA gene and two genetically unrelated control individuals	Gentamicin	The whole mitochondrial genome was amplified in a two-step PCR reaction and sequenced as previously described by Tanaka et al. (1996)	Susceptibility to aminoglycoside-induced toxicity	1) Mitochondrial glutathione was significantly reduced in the m.1095T>C mutant cybrids vs. controls. 2) The m.1095T>C cybrids also showed a 30% reduction in complex II/III activity 3) When challenged with 5 mg/mL gentamicin for 18 h, m.1095T>C mutant cybrids showed a 10-fold increase in annexin V positive/PI negative cells, signifying apoptotic cell death	1) NR /NR/ < 0.01 2) NR/NR/ < 0.013) NR/NR/ < 0.001
Pacheu-Grau et al. (2013)	Osteosarcoma 143B cybrids belonging to haplogroup H1, H but non-H1, J1, T, UK	Linezolid, paromomycin, chloramphenicol	Haplogroups were determined as previously described (Gómez-Durán et al., 2010, 2012). Three sequences were determined following the protocols from GeneChip® Human Mitochondrial Resequencing Array 2.0	Ribosomal antibiotic-induced adverse drug reactions	The amount of mitochondrial translation products, p.MT-CO1/SDHA ratio and the ratio of complex IV quantity to CS-specific activity were significantly higher in cybrids with the m.3010G (non-H1 and non-J1 haplogroup lineages) polymorphism compared with m.3010A (H1 and J1 haplogroup lineages) following treatment with linezolid. Therefore, m.3010G>A may be partially responsible for the side-effects associated with linezolid treatment in susceptible individuals	NR/NR/0.0386

*Abstraction table details the author(s), study design, drug(s) of interest, sequencing methods, outcomes, associations and statistics. CS, citrate synthase; cybrids, cytoplasmic hybrids; DILI, drug-induced liver injury; MT-COI, mitochondrially encoded cytochrome c oxidase subunit 1; NR, not reported; qPCR, quantitative polymerase chain reaction; rRNA, ribosomal RNA; SDHA, succinate dehydrogenase subunit A; SMRT, Single Molecule, Real-Time; SNP, single nucleotide polymorphisms; TB, tuberculosis*.

In agreement, an earlier article published by Pacheu-Grau et al. (2013) found that two 16S rRNA SNPs, m.2706 and m.3010, were responsible for the modulation of linezolid susceptibility in haplogroup H1 and J1 cybrids (Table 6). Specifically, the m.3010G>A polymorphism in the MT-RNR2 gene, characteristic of haplogroups H1/J1, results in a base pair modification in the 16S rRNA helix 48, leading to decreased synthesis of mtDNA-encoded polypeptides in cells treated with linezolid. Therefore, m.3010G>A may be partially responsible for the side-effects associated with linezolid treatment in susceptible individuals.

The association between mtDNA variations in the MT-RNR1 gene and sensorineural hearing loss in patients treated with aminoglycoside antibiotics (e.g., streptomycin, kanamycin and gentamicin) is one of the one of the most well-established links between a collection of mtDNA variants and the manifestation of adverse clinical outcomes, for a review refer to Barbarino et al. (2016). Individuals who carry *MT-RNR1* variants m.1555A>G and m.1494C>T are highly susceptible to ototoxicity regardless of dose, length of treatment or circulating serum concentration however the evidence surrounding the m.1095T>C variant has thus far been contentious (Barbarino et al., 2016).

A study published by Muyderman et al. (2012) aimed to demonstrate the pathogenicity of the m.1095T>C variant within an *in vitro* setting through the generation of three immortalized lymphoblastoid cybrid cell lines derived from an Italian individual harboring the m.1095T>C variant and two genetically unrelated control subjects. The authors showed that compared with control cybrids, m.1095T>C cybrids exposed to gentamicin (5 mg/mL) were more susceptible to apoptotic cell death, had greater caspase-3 activation (216 ± 16%, *p* < 0.01) and a 10-fold greater proportion of apoptotic cells (annexin V positive/PI negative cells, *p* < 0.001).

The final publication to investigate a putative association between mtDNA variation and adverse clinical outcomes from antimicrobial compounds was performed by Lee et al. (2019). Drug-induced liver injury (DILI) is the most severe side effect associated with antituberculosis medications such as isoniazid, rifampin or pyrazinamide. The study found that patients that presented with isoniazid-induced hepatotoxicity had more variants in complex I NADH subunit 1 (ND1; *p* = 0.025) and 5 (ND5; *p* = 0.028), a greater number of non-synonymous mutations in ND5 (1.5 ± 1.6 vs. 0.7 ± 0.8; *p* = 0.033) and a higher average ratio of non-synonymous to total substitutions compared with non-DILI controls. In contrast, no mtDNA associations were found in the patients presenting with rifampin- or pyrazinamide-induced liver injury. The authors hypothesized that variation in complex I subunits 1 and 5 may affect ETC function, thus predisposing susceptible individuals to isoniazid-induced DILI when inevitably exposed to the primary metabolite of isoniazid, hydrazine, which is a potent complex II inhibitor (Lee et al., 2019).

### Mitochondrial DNA Variants Associated With Altered Responses to Other Therapeutics

The remaining six articles that met the inclusion criteria covered a breadth of therapeutics for a variety of disease indications. Since the articles could not be grouped into a single medicine category, they were defined as “other” and will be discussed individually herein (Table 7).

**Table 7:** Summary of the publications included in this review that report differential responses to “other” medications.

**Author(s)**	**Study Description**	**Drug(s)**	**Sequencing Method**	**Outcome**	**Association**	**Odds Ratio/95% Confidence Intervals/*p*-value**
Di Lorenzo et al. (2009)	64 individuals with a history of migraines for at least 1 year and had completed an open trial with riboflavin (400 mg) with no concomitant drug treatment	Riboflavin (vitamin B2)	Mitochondrial haplogroups were determined by direct sequencing of the non-coding HVS-1 and HVS-2 regions of mtDNA. Patients were classified into two mtDNA haplogroups: “H” and “non-H”	Riboflavin efficacy in the treatment of migraines	Haplogroup H is a risk factor for treatment inefficacy Decrease in monthly attack frequency after riboflavin treatment was marginal in haplogroup H subjects	0.24/0.08–0.71/0.054.15/1.42–12.18/0.0001
Esteban et al. (2019)	91 patients with nAMD who received three initial monthly injections of intravitreal ranibizumab (0.5 mg/0.05 mL) followed by a flexible *pro re nata* period for 8 months	Ranibizumab	PCR with custom Taqman SNP genotyping assays for four mtDNA haplogroup markers (H: m.7028C>T; HV: m.14766T>C; JT: m.4216T>C; and U: m.12308A>G)	Response to intravitreal ranibizumab treatment in nAMD	After 4 months, BCVA scores were significantly higher for haplogroups HV, JT and U compared with baseline. CFT was significantly lower across all groups. At 12 months, JT BCVA scores were lower and CFT scores were higher than baseline	NR/NR/NRNR/NR/0.004
Guzmán-Fulgencio et al. (2014)	304 HIV/HCV coinfected patients who had completed a course of HCV therapy	PegIFN-α/ribavirin therapy	Genotyping was performed using the Sequenom MassARRAY platform iPLEX® Gold assay design system	SVR following pegIFN-α + ribavirin treatment	European mtDNA haplogroups were not related to HCV treatment response in HIV/HCV coinfected patients on pegIFN-α/ribavirin therapy	Multiple/Multiple/>0.05
Mittal et al. (2017)	113 European-Caucasian individuals selected from the CATIE study. Seventy four participants included after confounding factors removed	Risperidone, quetiapine or olanzapine	Mitochondrial genome was enriched by long-range PCR amplification. Products were subsequently purified and sequenced on the Illumina HiSeq 2500 platform	Antipsychotic-induced weight gain	No mtDNA SNPs were significantly associated with % weight change. No significant association between mtDNA phylogenetic groups (H-HV-V, J-T and K-U) and % weight change	NR/NR/NR
Subiabre et al. (2015)	191 warfarin-treated patients from a Chilean population, 91% of which had Amerindian background	Warfarin	Mitochondrial haplogroup characterization was performed using PCR and PCR restriction fragment length polymorphism techniques. Specific endonuclease digestion and gel visualization was used to determine the presence of 3 polymorphic sites in haplogroups A, C and D of the Amerindian population	Warfarin dosage requirements	There were no significant differences in warfarin dosage requirements among the different Amerindian haplogroups	NR/NR/0.083
Wang et al. (2019)	46 healthy peri/post-menopausal women with intact uteri and ovaries with at least one cognitive complaint and one vasomotor-related symptom	PhytoSERM (selective estrogen receptor β modulator)	Sequencing was performed by dye-terminator sequencing on a 96-capillary 3730xl DNA analyzer	Efficacy of phytoSERM for the management of menopausal symptoms	Haplogroup H had significantly deceased hot flash frequency when treated with 50 mg/day phytoSERM compared with placebo. Haplogroup H placebo subjects had lower baseline LOT scores compared with non-H haplogroups, 50 mg phytoSERM also prevented further decline in the haplogroup H	NR/NR/0.04NR/NR/0.007NR/NR/0.048

*Abstraction table details the author(s), study design, drug(s) of interest, sequencing methods, outcomes, associations and statistics. BVCA, best corrected visual acuity; CATIE, clinical antipsychotic trails of intervention effectiveness; CFT, central foveal thickness; HCV, hepatitis C virus; HIV, human immunodeficiency virus; LOT, learning over trials; mtDNA, mitochondrial DNA; nAMD, neovascular age-related macular degeneration; NR, not reported; PCR, polymerase chain reaction; pegIFN-α, pegylated-interferon-alpha; SNP, single nucleotide polymorphism; SVR, sustained virological response*.

A pilot study performed by Esteban et al. (2019), investigated the effect of mitochondrial haplogroups on response to the antivascular endothelial growth factor (VEGF) agent, ranibizumab, in neovascular age-related macular degeneration. Whilst the ethnicity of the cohort was not explicitly specified, patients were classified into four European haplogroups (HV, JT, U and “other”). Best corrected visual acuity (BCVA) scores were significantly higher upon treatment with ranibizumab in all haplogroups except the group defined as “other” after 4 months (HV: *p* = 0.021; JT: *p* = 0.002; U: *p* = 0.002). In addition, central foveal thickness (CFT) was significantly lower in all haplogroups (HV: *p* < 0.001; JT: *p* = 0.028; U: *p* = 0.043; Other: *p* = 0.007). However, no significant differences between groups were detected, likely due to small the sample size and insufficient statistical power. At 12 months, CFT values were different between groups (*p* = 0.004), and haplogroup JT showed lower BCVA than baseline and higher CFT values due to thickening after the intravitreal loading phase (*p* = 1.00), which may be suggestive of higher VEGF levels in haplogroup JT individuals, therefore indicating a requirement for higher and/or more frequent dosing with ranibizumab in these individuals (Esteban et al., 2019).

Pharmacogenomic research performed by Di Lorenzo et al. (2009) examined the influence of mtDNA haplogroups on riboflavin response in migraine sufferers (Table 7). Riboflavin appeared to be most effective in migraine sufferers of non-H mtDNA haplogroups whereas haplogroup H was a risk factor for treatment inefficacy. When examining migraine attack frequency between baseline and the fourth month of treatment, there was a marginal decrease in attack frequency in the haplogroup H patients (4.71 ± 3.85 to 3.22 ± 3.51, *p* = 0.05). In contrast, the non-H group showed a more pronounced decrease in attack frequency (4.43 ± 3.87 to 2.25 ± 2.01; *p* = 0.0001).

In a study performed by Guzmán-Fulgencio et al. (2014), the authors investigated the effects of mtDNA variation in relation to the efficacy of HCV therapeutic regimens. The article concluded that European mtDNA haplogroups were not significantly associated with the efficacy of pegylated-interferon-α (PegIFN-α) plus ribavirin for the treatment of HCV in HIV/HCV coinfected individuals. This remained the case regardless of stratification by HCV genotype or IL-28B (rs12980275) genotype.

Vitamin K antagonists such as warfarin are widely prescribed for the treatment of thromboembolic disease. However, there is a high degree of interindividual variation in dosage requirements amongst patients to achieve the desired anticoagulant effect (Subiabre et al., 2015). A study conducted by Subiabre et al. (2015) aimed to investigate the influence of ethnicity, defined by the presence of Amerindian haplogroups, on variability in warfarin treatment efficacy (Table 7). Results from the study showed that there were no significant differences in warfarin dosage requirements across Amerindian haplogroups (haplogroups A, B, C and D) in Chilean patients (*p* = 0.083).

Weight gain is a common and often severe side effect associated with the use of antipsychotic medications. An article by Mittal et al. (2017) examined the role of both nuclear-encoded mitochondrial genes and the mitochondrial genome in conferring risk for antipsychotic-induced weight gain in schizophrenic subjects. When performing linear regression with percentage weight change as the dependent variable and mitochondrial genotype as predictors, no SNPs were found to be significantly associated with weight change. In agreement, no major mitochondrial haplogroup lineages (H-HV-V, J-T or K-U) were associated with weight gain (Mittal et al., 2017).

Finally, Wang et al. (2019) performed retrospective analyses on a phase Ib/IIa clinical trial for phytoSERM, a selective estrogen receptor-beta modulator used for the treatment of menopausal symptoms, to identify potential treatment responders (Table 7). When stratifying patients by mitochondrial haplogroup, those belonging to haplogroup H had significantly fewer hot flash episodes when treated with 50 mg/day phytoSERM (PS50) compared with placebo (Median PS50: −1.64, Placebo: 0.43; *p* = 0.04). Those belonging to the haplogroup H placebo group had significantly decreased learning over trials (LOT) score during the clinical study compared with non-H haplogroups (Median H: −10, non-H: −2; *p* = 0.007). However, PS50 prevented further decline in the H haplogroup (Median PS50:−1, Placebo: −10; *p* = 0.048). No such preventative effect was observed in the non-H haplogroup. The authors concluded that mitochondrial haplogroup H may be a genetic indicator for phytoSERM treatment efficacy, though other associations should not be ruled out due to the small sample size of the study.

### Summary of Full-Text Articles Excluded From Review

A total of 37 full-text articles were excluded from review, a summary of these publications and their key findings are displayed in Supplementary Table 1. The most common reasons for article exclusion were that the publications addressed the effect of mtDNA variation upon health and disease rather than drug response (11 articles, 29.7%) or for reasons defined as “other” (11 articles, 29.7%). Examples of which include mitochondrial transplantation studies, population-wide genome sequencing studies or articles that examined mtDNA mutations associated with viral infection. Secondary to this, articles were excluded because they assessed the effects of mtDNA depletion rather than variation (6 articles, 16.2%) or examined chemical-induced, rather than drug-induced toxicity (4 articles, 10.8%). A small number of publications were excluded because they were reviews rather than original research articles (3 articles, 8.1%) or non-human models were used (2 articles, 5.4%).

## Discussion

The triad of drug efficacy, toxicity and resistance influence the risk-benefit balance of all therapeutics. The application of pharmacogenomics has the potential to improve the risk-benefit balance of a given therapeutic by enabling the stratification of patient populations, whereby efficacy is maximized and/or resistance and toxicity are minimized. To-date, pharmacogenomics has had an overwhelming focus on the nuclear genome; however, an increased understanding of the particulars of the mitochondrial genome, alongside increased availability of techniques for its interrogation, have provided the impetus to begin the expansion of pharmacogenomics to include this often-overlooked genome.

This systematic review identified 24 articles reporting an investigation of the association between mtDNA variant(s) and drug efficacy, toxicity or resistance. The diversity of mitochondrial variants studied in the articles included this review has prevented meta-analyses, particularly in the case of susceptibility to antiretroviral compounds, which are typically administered as part of an ART regimen; this means that ART-toxicities are not able to be linked to a specific drug or even drug class, and therefore studies cannot be compared like-for-like.

General conclusions may still be drawn however; haplogroup H was associated with greater ART efficacy compared with other European haplogroups in studies by Guzmán-Fulgencio et al. (2013) and Medrano et al. (2018), whereas haplogroup L2 was associated with diminished efficacy compared with non-L2 haplogroups in studies by Grady et al. (2011) and Hulgan et al. (2012). It should be noted however that haplogroups H and L2 were not directly compared in any of the articles included in this review, therefore it cannot be said that the efficacy of ART is superior in haplogroup H vs. haplogroup L2, only that each haplogroup displayed significantly different susceptibility to other haplogroups in the respective studies.

Despite being associated with superior efficacy in the aforementioned studies, haplogroup H has been associated with an increased risk of HAART-induced lipoatrophy, emphasizing the importance of considering all elements of the drug response triad (Hendrickson et al., 2009). The identification of mtDNA variants associated with ART-induced toxicity is convoluted further by an inability to distinguish between the phenotype of pathologies induced by HIV infection itself, and phenotypes that are a result of the toxicities associated with ART regimens. For example, peripheral neuropathy is a common neurological complication associated with HIV infection, occurring in up to 35% of patients, but it is also a known neurotoxic effect associated with ART therapy (Kampira et al., 2013a). This overlap makes it difficult to establish whether the mtDNA haplogroup is associated with susceptibility to mitochondrial dysfunction induced by HIV infection itself, or whether it is associated with susceptibility to ART-induced toxicity.

Antibiotic-induced mitochondrial toxicity has long been appreciated; the endosymbiotic origin of mitochondria has resulted in a protein synthesis apparatus similar to that of bacteria, and as a result, mitochondrial ribosomes are frequently unintended targets of antibiotics (Pacheu-Grau et al., 2013). Mitochondrial DNA variants have been studied as a potential source of toxicity on the basis that variants may confer altered antibiotic binding sites and therefore may modify susceptibility. Pacheu-Grau et al. (2013) reported increased susceptibility to linezolid-toxicity in cybrids harboring the m.3010G>A polymorphism. These findings were supported Garrabou et al. (2017) who found individuals harboring the m.3010G>A mutation to be more susceptible to adverse clinical outcomes from linezolid treatment. Conversely, Muyderman et al. (2012) demonstrated that cybrids containing the m.1095T>C mutation in *MT-RNR1* were more susceptible to aminoglycoside antibiotic-induced apoptotic cell death.

In addition to the studies of Pacheu-Grau et al. (2013) and Muyderman et al. (2012) a further three articles reported the use of cybrids to investigate the effect of mtDNA variants upon drug response (Sun et al., 2015; Patel et al., 2019; Abedi et al., 2020). Unlike human subjects, cybrids provide a controlled nuclear background upon which to study the effects of variants in the mitochondrial genome in isolation. The price for this controlled background however, is a lack of native, mito-nuclear communication, which is a significant contributor to the functional impact of mtDNA variants (Wilkins et al., 2014; Quirós et al., 2016).

When drawing conclusions from *in vitro* studies, one must also consider that the differences reported are at a cellular or even subcellular level; and whether this translates into a phenotypic effect at an organism level is not known. Such considerations were taken into account when evaluating the four articles in this review that reported the impact of mtDNA variants upon response to anticancer agents, where all articles reported the use of *in vitro* models. All studies sought to address the clinical problem of drug-resistance to anticancer agents, with greater sensitivity to cisplatin reported in haplogroups J (compared with haplogroup H), L and macrohaplogroup cluster A+B (compared with haplogroup D), while the m.6124T>C mutation was reported to confer resistance to simvastatin treatment (Sun et al., 2015; Patel et al., 2019; Abedi et al., 2020). In contrast, the fourth article reported that drug-sensitive and drug-resistant cell lines belonged to the same mitochondrial haplogroup i.e., the mitochondrial haplogroup did not confer drug sensitivity or resistance (Ma et al., 2015). Of the three articles reporting a difference conferred by haplogroup, speculative mechanisms were discussed, underpinned by experimental interrogations, including the assessment of mitochondrial membrane potential, gene expression and cellular levels of enzymes associated with ROS production (Sun et al., 2015; Patel et al., 2019; Abedi et al., 2020). In particular, Patel et al. (2019) deduced that differences between H vs. J GG stretch patterns in the mtDNA D-Loop may lead to differential formation of cisplatin-mtDNA adducts. Among all articles, there was an acknowledgment that the findings may not be evident *in vivo*. However, the use of *in vitro* models such as cybrids, in parallel with in-human studies would provide a more complete picture of both the phenotypic effect of any differences in mtDNA and the biological rationale of such effects, via unpicking the mechanism at a cellular level. Nonetheless, of the 24 articles included in this review, only two studies demonstrated the corroboration of *in vitro* model findings in human studies (see Pacheu-Grau et al., 2013; Garrabou et al., 2017 as discussed above).

Eleven of the articles included this review reported populations or haplogroups of European/Caucasian/White descent exclusively, including 4 of the 10 articles examining the association of mtDNA variation with differential response to ART (Hendrickson et al., 2009; Hulgan et al., 2011; Micheloud et al., 2011; Medrano et al., 2018). These four articles investigated associations of European haplogroups, which has limited global relevance as only 6% of people living with HIV are in Western/Central Europe and North America (unaids.org, 2020a,b). It should be noted however that five of the studies investigating ART response did observe associations with African haplogroups L0, L1, L2 and L3 in human studies; a particularly notable finding given the nuclear genome variation present in these studies (unlike cybrid studies) and the greater nuclear genome diversity in African populations compared with Asian and European populations (Yu et al., 2002; Campbell and Tishkoff, 2008). The majority of studies that did use a limited population were clear that the findings could not be extrapolated beyond the current population (Kampira et al., 2013b [Malawian]; Medrano et al., 2018 [Caucasian]).

Many articles studying the impact of mtDNA variation upon ART response reported a limited sample size and the associated effects on study power. In the case of Kampira et al. (2013a), this was due to performing a comparison between mitochondrial subhaplogroups (including L0a1 and L2a), rather than haplogroups. The result was an n number of between 3 and 32 per subhaplogroup. Had the authors compared between haplogroups, this would have resulted in an n number between 15 and 63, but at the cost of increased intragroup variation; haplogroups encompass much more variation than subhaplogroups, with the presence of many other SNPs beyond the select number of haplogroup-defining SNPs. This increased intragroup variation reduces the chance of identifying a significant association and certainly impairs the ability to detect less robust associations (Kampira et al., 2013a).

In summary, investigation of the impact of mtDNA variation upon drug response has been sporadic to-date and lags behind research into the nuclear genome. Although provocative, the collective assessment of associations reported in the articles included in this review are inconclusive due to heterogeneous methods and outcomes, limited racial/ethnic groups, lack of replication, and inadequate statistical power. Expectedly, no findings of mtDNA variants have resulted in a change in clinical practice. The authors recommend the following to increase robustness of the work in this field, such that where appropriate, findings may translate into clinical practice: (1) The parallel use of *in vitro* mechanistic models such as cybrids, in tandem with human association studies, (2) Recruitment of sufficient sample sizes at the subhaplogroup level in order to reduce intragroup variation while retaining sufficient statistical power (3) Increased publications where no association has been reported, to prevent the repetition of experiments and the consequent delay in genuine progress in the field. Recruitment in-line with previously-reported associations would also be of great benefit in either the replication or refutation of current findings. There was a general consensus across the articles reviewed that the application of mitochondrial genomics to identify susceptible individuals is not a magic bullet, and this is indeed true; drug response is affected by a complex array of elements, but so long as there remains a high degree of idiosyncrasy in drug response, further research in this field is likely to be of clinical benefit when considered in the context of additional susceptibility factors.

## Data Availability Statement

The original contributions presented in the study are included in the article/Supplementary Material, further inquiries can be directed to the corresponding author.

## Author Contributions

SJ and AB contributed equally to the compilation and review of this work. AC and AA contributed equally in their critical appraisal. All authors contributed to the article and approved the submitted version.

## Conflict of Interest

The authors declare that the research was conducted in the absence of any commercial or financial relationships that could be construed as a potential conflict of interest.

## Publisher's Note

All claims expressed in this article are solely those of the authors and do not necessarily represent those of their affiliated organizations, or those of the publisher, the editors and the reviewers. Any product that may be evaluated in this article, or claim that may be made by its manufacturer, is not guaranteed or endorsed by the publisher.
