# Supplementary material for: The Role of Mitochondrial DNA Variation in Drug Response: A Systematic Review
Source: Front Genet. 2021 Aug 17;12:698825. doi: 10.3389/fgene.2021.698825 (PMC8416105; doi:10.3389/fgene.2021.698825)
Supplement: Supplementary file 1 [file Table_1.DOCX]

Supplementary Table 1 Studies are described with reasons for exclusion: (1) Publication is not a research article, (2) Publication assesses the effect of mtDNA depletion rather than mtDNA variation, (3) Publication uses non-human models, (4) Publication assesses the effect of mtDNA variation on disease/health rather than drug response, (5) Publication assesses the effect of mtDNA variation on chemical-induced rather than drug-induced toxicity, (6) Other.

| Author | Year | Summary | Reason for Exclusion |
| --- | --- | --- | --- |
| Al-Malkey *et al* | 2013 | Case report describing a pediatric patient harboring the m.1555A>G ototoxicity risk mutation with well-preserved hearing despite exposure to aminoglycoside antibiotics. | 1 |
| Amo *et al* | 2017 | Cisplatin resistance is driven by shorter mtDNA oriB (T16189C) variants in HeLa cybrids with healthy mitochondria. | 6 |
| Arbini *et al* | 2013 | MtDNA depletion sensitizes cancer cells to PARP inhibitors though BRCA2 repression. | 2 |
| Cacabelos and Martinez-Bouza | 2011 | Review of the incorporation of pharmacogenomic procedures to optimize schizophrenia therapeutics. | 1 |
| Chang *et al* | 2019 | Mitochondrial transplantation has antitumor potential in breast cancer. | 4 |
| Datta *et al* | 2017 | Benzalkonium chloride inhibits mitochondrial complex I. Cells bearing LHON mtDNA mutations are more sensitive to BAK toxicity. | 5 |
| Ding *et al* | 2017 | Nine mt-tRNA mutations were identified in patients with polycystic ovary syndrome presenting with insulin resistance. | 4 |
| Dolinko *et al* | 2020 | Mitochondria from African and Asian diabetic subjects possess a ‘metabolic memory’ that confers resistance against hyperglycemia, hypoxia and demethylation. | 4 |
| Elliott and Al-Hajj | 2009 | ATP-binding cassette protein, ABCB8 mediates doxorubicin resistance in melanoma cells by protecting the mitochondrial genome. | 6 |
| Ghelli *et al* | 2009 | Mitochondrial haplogroup J increases the sensitivity of Leber's hereditary optic neuropathy cells to 2,5-hexanedione toxicity. | 5 |
| Girolimetti *et al* | 2017 | Platinum-induced mtDNA mutations confer chemoresistance to the anticancer agent, paclitaxel. | 6 |
| Gonzalez-Sanchez *et al* | 2014 | MtDNA depletion may activate mechanisms of chemoresistance, hindering the efficacy of chemotherapy against hepatocellular carcinoma. | 2 |
| Gupta *et al* | 2012 | The first sequencing of a genome from the state of Kerala, India. | 6 |
| Hart *et al* | 2013 | A systematic review of mitochondrial haplogroups and outcomes of HIV infection and antiretroviral therapy. | 1 |
| Jiang *et al* | 2013 | MtDNA depletion and respiratory chain defects play critical roles in the pathogenesis of kidney injury induced by aristolochic acid I. | 2 |
| Jimenez-Sousa *et al* | 2015 | Cluster HV and haplogroup H are associated with decreased odds of severe sepsis among patients have undergone cardiac surgery. | 4 |
| Kallianpur and Hulgan | 2009 | A review of the pharmacogenetics of NRTI-associated peripheral neuropathy. | 1 |
| Koh *et al* | 2017 | MtDNA MT-CO1 variant was present in H1975 lung cancer cells (EGFR T790M, mutants) with acquired resistance to the PI3K/mTOR inhibitor, BEZ235. Depletion of mtDNA in parental cell line induces resistance to BEZ235. | 2 |
| Kuo *et al* | 2016 | Diabetes-susceptible (B4) and protective (D4) mitochondrial haplogroups have differential mitochondrial dynamics. | 4 |
| Li *et al* | 2017 | HIV-infected, antiretroviral therapy-experienced patients with and without toxicity have higher than average numbers of mtDNA variants than uninfected controls. | 4 |
| Lin *et al* | 2019 | The transfer of healthy mitochondria from Wharton's jelly mesenchymal stem cells to rotenone-stressed fibroblasts from a MELAS patient eliminates the mutational burden and rescues mitochondrial function. | 6 |
| Ma *et al* | 2014 | Initial evidence for an association between host ancestral genome and the structure of its microbiome. | 4 |
| Malik *et al* | 2014 | Cellular response to sub-lethal UV radiation is differential between mitochondrial haplogroup H and J cybrids. | 4 |
| Marin *et al* | 2013 | Mitochondrial genome depletion in human liver cells abolishes bile acid-induced apoptosis. | 4 |
| Mizutani *et al* | 2009 | Mutant cybrids are more resistant to anticancer drugs, staurosporine, 5-fluorouracil and cisplatin than their wild-type counterparts when transplanted into mice. | 3 |
| Nishimura and Watanuki | 2014 | Haplogroup D volunteers experience greater non-shivering thermogenesis than non-haplogroup D volunteers. | 4 |
| Obara-Moszynska *et al* | 2013 | A case study: A large mtDNA deletion had no impact on response to recombinant growth hormone treatment. | 6 |
| Ouyang *et al* | 2016 | Findings suggest that mtDNA mutations occur frequently in long-term antiretroviral-treated, HIV-1-infected children. | 6 |
| Ouyang *et al* | 2018 | Longitudinal sampling showed a positive correlation between mtDNA mutations and treatment time in PBMCs from HIV-1-infected children on antiretroviral therapy. | 6 |
| Perez *et al* | 2011 | Mitochondrial genome depletion dysregulates bile acid- and paracetamol-induced expression of the transporters Mdr1, Mrp1 and Mrp4 in liver cells. | 2 |
| Prabhu *et al* | 2013 | Resveratrol depletes mtDNA. Inhibition of autophagy enhances resveratrol-induced caspase activation. | 2 |
| Strobbe *et al* | 2018 | Haplogroup J1 is more sensitive to the pesticide rotenone whereas haplogroup K1 is more resistant. | 5 |
| Terrazzino *et al* | 2016 | Mitochondrial haplogroup H has been found to be protective in the development of radiation-induced fibrosis in breast cancer treatment. | 5 |
| Thaker *et al* | 2016 | Cybrids with haplogroup K have increased expression of ApoE and protection from amyloid-beta toxicity. | 4 |
| Vo *et al* | 2018 | Cybrids generated from patients with age-related macular degeneration and age-matched controls show decreased latex bead uptake when treated with anti-VEGF drugs. | 6 |
| Wu *et al* | 2012 | During aging in mice, the oxidative stress-related increase of the adaptor protein, p66Shc and Ser36-P-p66Shc (serine36-phosphorylated) may be associated with the accumulation of the mtDNA 3873-bp deletion in the inner ear. | 3 |
| Zhang *et al* | 2016 | Mitochondrial biogenesis and aberrant bioenergetics are potential therapeutic targets to overcome drug resistance to mitogen-activated protein kinase inhibitors. | 6 |

Abbreviations: ApoE, apolipoprotein E; bp, base pair; EGFR, epidermal growth factor receptor; HIV, human immunodeficiency virus; MELAS, mitochondrial encephalopathy, lactic acidosis, and stroke-like episodes; MT-CO1, mitochondrially encoded cytochrome c oxidase subunit; mtDNA, mitochondrial DNA; NRTI, nucleoside reverse transcriptase inhibitor; PARP, Poly (ADP-ribose) polymerase; PBMC, peripheral blood mononuclear cell.
